# Incidental hemobilia diagnosed during emergency ERCP for acute cholangitis in a patient on antiplatelet therapy

**DOI:** 10.1093/jscr/rjag391

**Published:** 2026-05-24

**Authors:** Songming Ding, Yang Liu, Yingying Su, Shusen Zheng, Qiyong Li

**Affiliations:** Division of Hepatobiliary and Pancreatic Surgery, Shulan (Hangzhou) Hospital Affiliated to Zhejiang Shuren University, Shulan International Medical College, No. 848 Dongxin Road, Hangzhou 310003, P.R. China; Endoscopy Center, Shulan (Hangzhou) Hospital Affiliated to Zhejiang Shuren University, Shulan International Medical College, No. 848 Dongxin Road, Hangzhou 310003, P.R. China; Endoscopy Center, Shulan (Hangzhou) Hospital Affiliated to Zhejiang Shuren University, Shulan International Medical College, No. 848 Dongxin Road, Hangzhou 310003, P.R. China; Division of Hepatobiliary and Pancreatic Surgery, Shulan (Hangzhou) Hospital Affiliated to Zhejiang Shuren University, Shulan International Medical College, No. 848 Dongxin Road, Hangzhou 310003, P.R. China; Division of Hepatobiliary and Pancreatic Surgery, Shulan (Hangzhou) Hospital Affiliated to Zhejiang Shuren University, Shulan International Medical College, No. 848 Dongxin Road, Hangzhou 310003, P.R. China

**Keywords:** hemobilia, acute cholangitis, choledocholithiasis, endoscopic retrograde cholangiopancreatography, antiplatelet therapy

## Abstract

Antiplatelet therapy is a well-established risk factor for gastrointestinal bleeding, yet hemobilia induced by these agents remains rarely reported. Acute cholangitis secondary to choledocholithiasis in patients on antiplatelet therapy presents significant clinical challenges, as the medication can precipitate hemobilia, subsequently exacerbating the cholangitis. Here, we report a 73-year-old female with a history of multiple biliary interventions and long-term aspirin use, exemplifying this clinical challenge. Although elective endoscopic retrograde cholangiopancreatography (ERCP) was scheduled following a 1-week aspirin discontinuation, the sudden onset of severe abdominal pain necessitated emergency intervention. ERCP revealed hemobilia with clots obstructing the major duodenal papilla. Therapeutic ERCP successfully evacuated the clots, extracted the stones, and placed a nasobiliary drain. The patient stabilized without requiring angiography or surgery. This case highlights that abrupt changes in abdominal pain patterns in calculous cholangitis patients receiving antiplatelet therapy should raise suspicion for hemobilia, underscoring the need for timely and effective clinical intervention.

## Introduction

Hemobilia, defined as bleeding from the biliary tree into the gastrointestinal tract, was first described in 1654 [1]. Although rare, it is potentially fatal if not promptly diagnosed and treated [2]. Notably, its incidence has risen in recent years, paralleling the increased frequency of hepatopancreatobiliary interventions [3]. However, establishing a timely diagnosis remains challenging. The complete classic triad of upper gastrointestinal hemorrhage, biliary colic, and jaundice manifests in only a minority of cases. Furthermore, the appearance of these signs often lags significantly behind the actual onset of biliary bleeding, leading to dangerous delays in recognition [4, 5]. Here, we present a case of a patient on antiplatelet therapy in whom hemobilia was unexpectedly identified during emergency endoscopic retrograde cholangiopancreatography (ERCP) performed for gallstone-related cholangitis.

## Case presentation

A 73-year-old female with a complex hepatobiliary history—including prior cholecystectomy, partial hepatectomy, two common bile duct (CBD) explorations, and multiple ERCPs (most recent 43 months prior)—presented with a 2-week history of low-grade fever. Her medical history was significant for carotid artery stenting, for which she had been on lifelong aspirin therapy (100 mg daily). On admission, she was febrile (38.0°C) but denied abdominal pain, jaundice, or signs of gastrointestinal bleeding.

**Figure 1:**
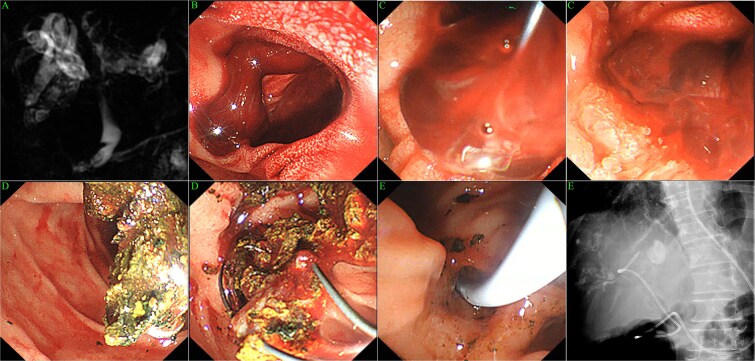
Diagnostic imaging and endoscopic management of incidental hemobilia. (A) Magnetic resonance cholangiopancreatography (MRCP) demonstrating diffuse intrahepatic ductal dilation and multifocal calculi, coexisting with a hilar stricture and choledocholithiasis. (B) Endoscopic view of the major duodenal papilla obstructed by fresh blood clots, indicating active hemobilia. (C) Endoscopic evacuation of blood clots using a retrieval basket. (D) Endoscopic extraction of common bile duct (CBD) stones. (E) Placement of an endoscopic nasobiliary drainage (ENBD) catheter for biliary decompression and monitoring.

Laboratory investigations indicated an acute inflammatory response and cholestasis: C-reactive protein was elevated at 53.8 mg/L (normal <5.0 mg/L), heparin-binding protein at 19.6 ng/ml (normal <15.0 ng/L), alkaline phosphatase at 424 U/L (normal 50–135 U/L), and γ-glutamyl transpeptidase at 213 U/L (normal 7–45 U/L). Hemoglobin was 115 g/L (normal 115–150 g/L). Magnetic resonance cholangiopancreatography revealed diffuse intrahepatic biliary dilation associated with multiple calculi, complicated by a hilar stricture and concurrent choledocholithiasis (Fig. 1A). These findings were consistent with a diagnosis of acute calculous cholangitis.

Antibiotics were initiated, and an elective ERCP was scheduled following a planned 7-day cessation of aspirin. However, the clinical course changed abruptly the following day when the patient developed sudden, severe right upper quadrant pain requiring potent analgesia (tramadol). An emergency ERCP was immediately performed. Upon entering the duodenum, the endoscopist observed blood-stained mucosa and fresh blood clots obstructing the major duodenal papilla (Fig. 1B). Therapeutic ERC was undertaken to evacuate blood clots and extract CBD stones using a retrieval basket, followed by the placement of an endoscopic nasobiliary drainage (ENBD) catheter (Fig. 1C–E and Supplementary Video 1). Post-procedure, the ENBD catheter drained clear bile within 12 h, indicated spontaneous cessation of bleeding. The patient was discharged on day 6 with stable hemoglobin levels (>102 g/L) after declining further invasive interventions, such as percutaneous transhepatic cholangioscopy.

## Discussion

Hemobilia, defined as hemorrhage originating from the liver, extrahepatic bile ducts (including the gallbladder), or the pancreas [6], was first described by Francis Glisson during the autopsy of a nobleman who succumbed to a fatal stab wound sustained in a fencing duel [7]. Over time, the etiology of this condition has undergone a significant transformation. Currently, iatrogenic hepatopancreatobiliary interventions represent the predominant cause, while other etiologies encompass cholelithiasis, vascular malformations, and neoplastic factors [8]. Meanwhile, antiplatelet therapy has been recognized as a significant risk factor; however, the most frequently reported cases involve antiplatelet drug-induced gallbladder hemorrhage [9, 10]. Notably, instances of antiplatelet agent-induced hemobilia occurring in the context of cholelithiasis-associated cholangitis are rarely documented. Furthermore, although hemobilia has a classic clinical presentation—Quincke’s triad (gastrointestinal bleeding, right upper quadrant pain, and jaundice)—the complete triad is observed in only 13.5% to 22% of patients [8]. Therefore, diagnosing atypical cases remains challenging, often leading to significant delays or incidental discovery [4, 11].

Our case exemplifies this diagnostic dilemma. Although the classic triad was absent on admission, the convergence of recurrent biliary interventions, antiplatelet therapy, and acute cholangitis—particularly the alteration in pain character [12]—should have raised early suspicion for hemobilia. Maintaining a high index of suspicion is vital to prevent diagnostic delays and ensure prompt intervention rather than reactive crisis management.

Management strategies range from conservative support to endoscopic, angiographic, or surgical interventions [1, 13]. ERCP offers a distinct advantage by serving dual diagnostic and therapeutic roles, allowing for direct visualization, clot removal, and drainage. In our patient, clearing biliary clots and stones combined with nasobiliary drainage effectively resolved the hemorrhage and controlled cholangitis, obviating the need for invasive modalities like transcatheter arterial embolization or surgery.

In conclusion, clinicians should maintain high vigilance for hemobilia in patients on antiplatelet therapy presenting with calculous cholangitis, particularly when there are significant changes in abdominal pain patterns. The minimally invasive endoscopic approach proved effective in this case, preventing severe complications and avoiding more aggressive interventions. Nevertheless, because this is a report of a single case, additional reports and research are needed to further understand the underlying mechanisms and to develop evidence-based clinical decision-making strategies.

## Supplementary Material

rjag391_Video_1
